# Eﬄux pump-mediated drug resistance in *Burkholderia*

**DOI:** 10.3389/fmicb.2015.00305

**Published:** 2015-04-14

**Authors:** Nicole L. Podnecky, Katherine A. Rhodes, Herbert P. Schweizer

**Affiliations:** ^1^Department of Microbiology, Immunology and Pathology, College of Veterinary Medicine and Biological Sciences, Colorado State UniversityFort Collins, CO, USA; ^2^Department of Molecular Genetics and Microbiology, College of Medicine, Emerging Pathogens Institute, Institute for Therapeutic Innovation, University of FloridaGainesville, FL, USA

**Keywords:** *Burkholderia*, antibiotics, resistance, eﬄux pump, adaptation

## Abstract

Several members of the genus *Burkholderia* are prominent pathogens. Infections caused by these bacteria are difficult to treat because of significant antibiotic resistance. Virtually all *Burkholderia* species are also resistant to polymyxin, prohibiting use of drugs like colistin that are available for treatment of infections caused by most other drug resistant Gram-negative bacteria. Despite clinical significance and antibiotic resistance of *Burkholderia* species, characterization of eﬄux pumps lags behind other non-enteric Gram-negative pathogens such as *Acinetobacter baumannii* and *Pseudomonas aeruginosa*. Although eﬄux pumps have been described in several *Burkholderia* species, they have been best studied in *Burkholderia cenocepacia* and *B. pseudomallei*. As in other non-enteric Gram-negatives, eﬄux pumps of the resistance nodulation cell division (RND) family are the clinically most significant eﬄux systems in these two species. Several eﬄux pumps were described in *B. cenocepacia*, which when expressed confer resistance to clinically significant antibiotics, including aminoglycosides, chloramphenicol, fluoroquinolones, and tetracyclines. Three RND pumps have been characterized in *B. pseudomallei*, two of which confer either intrinsic or acquired resistance to aminoglycosides, macrolides, chloramphenicol, fluoroquinolones, tetracyclines, trimethoprim, and in some instances trimethoprim+sulfamethoxazole. Several strains of the host-adapted *B. mallei*, a clone of *B. pseudomallei*, lack AmrAB-OprA, and are therefore aminoglycoside and macrolide susceptible. *B. thailandensis* is closely related to *B. pseudomallei*, but non-pathogenic to humans. Its pump repertoire and ensuing drug resistance profile parallels that of *B. pseudomallei*. An eﬄux pump in *B. vietnamiensis* plays a significant role in acquired aminoglycoside resistance. Summarily, eﬄux pumps are significant players in *Burkholderia* drug resistance.

## The Genus Burkholderia

The genus *Burkholderia* comprises metabolically diverse and adaptable Gram-negative bacteria that are able to thrive in different, often adversarial, environments. Their metabolic versatility and adaptability is in part due to the coding capacity provided by large (7–9 Mb) genomes consisting of several chromosomes and in some species, e.g., *Burkholderia cenocepacia*, plasmids (Holden et al., 2004, 2009; Agnoli et al., 2012). Many members of the genus are clinically significant pathogens with renowned virulence potential (Tegos et al., 2012) and drug resistance (Burns, 2007). In contrast to most other Gram-negative pathogens, *Burkholderia* species are intrinsically polymyxin resistant and therefore colistin cannot be used as drug of last resort (Loutet and Valvano, 2011). Despite clinical significance and recognized antibiotic resistance of *Burkholderia* species, characterization of eﬄux pumps lags significantly behind other non-enteric Gram-negative pathogens such as *Acinetobacter baumannii* and *Pseudomonas aeruginosa* (Nikaido and Pages, 2012). Many *Burkholderia* eﬄux systems have homologs in other Gram-negatives, including *A. baumannii* and *P. aeruginosa*, and it is now generally believed that the multidrug resistance exhibited by these opportunistic pathogens is largely attributable to the existence of similar eﬄux pumps in these organisms (Poole, 2001; McGowan, 2006). As with other Gram-negative bacteria, the relative roles that individual eﬄux pumps play in intrinsic or acquired antibiotic resistance in the respective *Burkholderia* species are in many instances difficult to discern for various reasons: (1) a subset of the pumps found in any organism usually exhibits a considerable degree of substrate promiscuity, i.e., they recognize and extrude chemically and structurally diverse compounds, which leads to similar multidrug resistance profiles; (2) many of the eﬄux systems are not expressed at significant levels in wild-type strains under laboratory conditions and there exists a significant knowledge gap regarding the environmental conditions under which eﬄux genes are expressed; and (3) well characterized clinical or laboratory isolates expressing or lacking the respective eﬄux pumps often do not exist or are difficult to obtain (Mima and Schweizer, 2010; Coenye et al., 2011; Biot et al., 2013; Buroni et al., 2014). In this review we will summarize the current state of knowledge of eﬄux pumps and their roles in antibiotic resistance in the genus *Burkholderia,* which have been characterized to various degrees in a few representative organisms.

### Burkholderia cepacia Complex

The *Burkholderia cepacia* complex (BCC) currently comprises 17 closely related species (Mahenthiralingam et al., 2005; Vanlaere et al., 2009; Vandamme and Dawyndt, 2011). Some BCC members exhibit beneficial aspects such as use in biocontrol, a practice that has since been abandoned because of the risk of infection of compromised individuals (Kang et al., 1998). Many are opportunistic pathogens, being particularly problematic for cystic fibrosis patients and immune compromised individuals. *B. cenocepacia* and *B. multivorans* account for 85% of all BCC infections (Drevinek and Mahenthiralingam, 2010). BCC infections are difficult to treat because of intrinsic antibiotic resistance and persistence in the presence of antimicrobials (Golini et al., 2004; Peeters et al., 2009; Rajendran et al., 2010; Jassem et al., 2011). *B. vietnamiensis* belongs to the BCC group and sporadically infects cystic fibrosis patients (Jassem et al., 2011).

### Burkholderia pseudomallei

*Burkholderia pseudomallei* is a saprophyte and opportunistic pathogen endemic to tropical and subtropical regions of the world, and recent studies suggest that is more widespread than previously thought (Wiersinga et al., 2006, 2012, 2015; Currie et al., 2010). Since the U.S. anthrax attacks in 2001 the bacterium has received increasing attention because of its biothreat potential (Cheng et al., 2005), a history dating back to its use with malicious intent in a Sherlock Holmes short story (Vora, 2002). In the U.S. it is a strictly regulated Tier 1 select agent, which must be handled in approved biosafety level 3 (BSL-3) laboratories. The bacterium is the etiologic agent of melioidosis, a difficult-to-treat multifacteted disease (Wiersinga et al., 2006, 2012). The disease affects at-risk patients, including those suffering from cystic fibrosis (Schulin and Steinmetz, 2001; Holland et al., 2002), non-cystic fibrosis bronchiectasis (Price et al., 2013), and diabetes (Simpson et al., 2003). *B. pseudomallei* infections are recalcitrant to antibiotic therapy because of the bacterium’s intrinsic resistance due to expression of resistance determinants such as β-lactamase and eﬄux pumps, as well as contributing factors such as both intracellular and biofilm lifestyles (Schweizer, 2012b).

### Burkholderia mallei

*Burkholderia mallei* is an obligate zoonotic pathogen and the etiologic agent of glanders, which has been used as a bioweapon (Cheng et al., 2005; Whitlock et al., 2007). This bacterium likely diverged from *B. pseudomallei* upon introduction into an animal host approximately 3.5 million years ago (Losada et al., 2010; Song et al., 2010). The ensuing in-host evolution through massive expansion of insertion (IS) elements, IS-mediated gene deletion, and genome rearrangement, and prophage elimination is likely also responsible for the generally increased antibiotic susceptibility of *B. mallei* when compared to *B. pseudomallei,* presumably due to inactivation of resistance determinants (Nierman et al., 2004).

### Burkholderia thailandensis

*Burkholderia thailandensis* is closely related to *B. pseudomallei* (Brett et al., 1998). Although *B. thailandensis* has sporadically been shown to cause human disease (Glass et al., 2006), it is generally considered non-pathogenic and has often been used as a surrogate for antimicrobial compound and vaccine efficacy studies because the bacterium can be handled at BSL-2. Some strains are more closely related to *B. pseudomallei* than others. For instance, unlike the widely used *B. thailandensis* prototype strain E264 (Brett et al., 1998), strain E555 expresses the same capsular polysaccharide as *B. pseudomallei* (Sim et al., 2010). Since capsular polysaccharide is a potent immunogen this similarity was exploited in a vaccine study, which showed that immunization with live cells of this avirulent strain protects mice from challenge with a virulent *B. pseudomallei* strain (Scott et al., 2013).

## Burkholderia cenocepacia

### Eﬄux Pumps and Drug Resistance

Early reports provided mostly indirect evidence that *B. cenocepacia* eﬄux pumps play a role in drug eﬄux. The synergy between reduced outer membrane permeability and eﬄux was cited as a common theme of the increased resistance that non-fermenting Gram-negative bacteria like *A. baumannii*, *P. aeruginosa*, and *B.* (*ceno*)*cepacia* display (Hancock, 1998). An analysis of the DsbA–DsbB disulfide bond formation system revealed that *dsbA* and *dsbB* mutation resulted increased susceptibilities to a variety of antibiotics (Hayashi et al., 2000). This led to the conclusion that the DsbA–DsbB system might be involved in the formation of a multidrug resistance system. Another early report described an outer membrane lipoprotein involved in multiple antibiotic (chloramphenicol, trimethoprim, and ciprofloxacin) resistance (Burns et al., 1996). This protein, OpcM, is the outer membrane channel of an eﬄux pump of the resistance nodulation cell division (RND) family that was subsequently named CeoAB-OpcM, which was shown to be inducible by salicylate and chloramphenicol (Nair et al., 2004). Eﬄux was also shown early on to play a role in fluoroquinolone resistance (Zhang et al., 2001).

Genome analysis and homology searches led to identification of an additional 14 open reading frames encoding putative components RND family eﬄux pumps (Guglierame et al., 2006). A summary of pertinent features of at least partially characterized *B. cenocepacia* RND eﬄux pumps and their relationships to RND systems in other *Burkholderia* species is presented in **Table 1**.

**Table 1:** Partially characterized resistance nodulation cell division (RND) eﬄux pumps in *Burkholderia* species.

Species	Eﬄux pump	Gene names	Gene annotation	Major substrates	Reference
*Burkholderia cenocepacia*					
	RND-1	NA	BCAS0591–BCAS0593	Non-detectable	Buroni et al. (2009)
	RND-3	NA^1^	BCAL1674–BCAL1676	Nalidixic acid, ciprofloxacin, tobramycin, chlorhexidine^3^	Buroni et al. (2009, 2014), Coenye et al. (2011)
	RND-4	NA^2^	BCAL2820–BCAL2822	Aztreonam, chloramphenicol, fluoroquinolones, tobramycin	Bazzini et al. (2011b)
	RND-8	NA	BCAM0925–BCAM0927	Tobramycin^3^	Buroni et al. (2014)
	RND-9	NA	BCAM1945–BCAM1947	Tobramycin^3^, chlorhexidine^3^	Coenye et al. (2011), Buroni et al. (2014)
	RND-10	*ceoAB-opcM*^4^	BCAM2551–BCAM2549	Chloramphenicol, fluoroquinolones, trimethoprim	Nair et al. (2004)
*B. pseudomallei*					
	AmrAB-OprA	*amrAB-oprA*	BPSL1804–BPSL1802	Aminoglycosides, macrolides, cethromycin	Moore et al. (1999), Mima et al. (2011)
	BpeAB-OprB	*bpeAB-oprB*	BPSL0814–BPSL0816	Chloramphenicol, fluoroquinolones, macrolides, tetracyclines^5^	Chan et al. (2004), Mima and Schweizer (2010)
	BpeEF-OprC	*bpeEF-oprC*	BPSS0292–BPSS0294	Chloramphenicol, fluoroquinolones, tetracyclines, trimethoprim^6^	Kumar et al. (2006), Schweizer (2012a,b)
*B. thailandensis*					
	AmrAB-OprA	*amrAB-oprA*	BTH_I2445–BTH_I2443	Aminoglycosides, macrolides, tetracyclines	Biot et al. (2013)
	BpeAB-OprB	*bpeAB-oprB*	BTH_I0680–BTH_I0682	Tetracyclines	Biot et al. (2013)
	BpeEF-OprC	*bpeEF-oprC*	BTH_II2106–BTH_II2104	Chloramphenicol, fluoroquinolones, tetracyclines, trimethoprim/sulfamethoxazole	Biot et al. (2011, 2013)
*B. vietnamiensis*					
	AmrAB-OprM	*amrAB-oprM*	Bcep1808_1574–Bcep1808_1576	Aminoglycosides	Jassem et al. (2014)

*NA, not applicable*

*^1^Corresponds to B. pseudomallei amrAB-oprA*

*^2^Corresponds to B. pseudomallei bpeAB-oprB*

*^3^Sessile (biofilm grown) cells only*

*^4^Corresponds to B. pseudomallei bpeEF-oprC*

*^5^Low-level resistance in de-repressed (ΔbpeR) strains*

*^6^High-level resistance in regulatory mutants, e.g., bpeT carboxy-terminal mutations*

Expression of one of these, orf2, in *Escherichia coli* conferred resistance to several antibiotics (Guglierame et al., 2006). The roles of several RND transporters – RND-1, RND-3, and RND-4 – in intrinsic *B. cenocepacia* drug resistance was subsequently assessed by mutational analyses, which showed that RND-3 and RND-4, but not RND-1, contribute to *B. cenocepacia*’s intrinsic resistance to antibiotics and other inhibitory compounds (Buroni et al., 2009). A subsequent study comparing RND-4 and RND-9 single and double mutants confirmed the role that RND-4 plays in *B. cenocepacia*’s antibiotic resistance and also showed that RND-9 contributed only marginally to resistance (Bazzini et al., 2011b). Completion of the strain J2315 genome sequence showed that it encodes 16 RND eﬄux systems, which provides evidence for the biological relevance of these transporters in this bacterium and also enables global analyses of RND pump expression (Holden et al., 2009; Perrin et al., 2010; Bazzini et al., 2011a; Buroni et al., 2014). For example, deletion of the 16 putative RND operons from *B. cenocepacia* strain J2315 showed that these pumps play differential roles in the drug resistance of sessile (biofilm) and planktonic cells. These studies revealed that: (1) RND-3 and RND-4 play important roles in resistance to various antibiotics, including ciprofloxacin and tobramycin, in planktonic populations; (2) RND-3, RND-8, and RND-9 protect from the antimicrobial effects of tobramycin in biofilm cells; and (3) RND-8 and RND-9 do not play a role in ciprofloxacin resistance (Buroni et al., 2014). An emerging theme from these studies is that RND-3 seems to play a major role in *B. cenocepacia*’s intrinsic drug resistance. It was suggested that mutations in the RND-3 regulator-encoding gene may be responsible for this pump’s prevalent overexpression and accompanying high-level antibiotic resistance in clinical BCC isolates (Tseng et al., 2014).

Studies aimed at assessing chlorhexidine mechanisms in *B. cenocepacia* J2315 confirmed the differential roles that RND pumps play in biofilm versus planktonically grown cells. RND-4 contributed to chlorhexidine resistance in planktonic cells, whereas RND-3 and RND-9 played a role in chlorhexidine resistance in sessile cells (Coenye et al., 2011). Mutational analyses of 2-thiopyridine resistant mutants showed that RND-4 confers resistance to an anti-tubercular 2-thiopyridine derivative (Scoffone et al., 2014). The involvement of eﬄux pumps in tigecycline resistance was inferred from the potentiating effects of the eﬄux pump inhibitor (EPI) MC-207,110 on tigecycline’s anti-*B. cenocepacia* activity (Rajendran et al., 2010).

Eﬄux pumps belonging to other families may also contribute to *B. cenocepacia*’s drug resistance. Experiments with an immunodominant antigen in cystic fibrosis patients infected with *B. cenocepacia* identified a drug eﬄux pump, BcrA, which is a member of the major facilitator superfamily (MFS). It was shown to confer tetracycline and nalidixic acid resistance when expressed in *E. coli* (Wigfield et al., 2002). Upregulation of an eﬄux pump resulted in resistance to the phosphonic acid antibiotic fosfidomycin (Messiaen et al., 2011). This pump is a homolog of Fsr, a member of the MFS, which was previously shown to confer fosmidomycin resistance on *E. coli* (Fujisaki et al., 1996; Nishino and Yamaguchi, 2001).

### Other Functions of *B. cenocepacia* RND Eﬄux Pumps

As with other Gram-negative bacteria, the function of *B. cenocepacia* eﬄux pumps extends beyond antibiotic resistance. In *B. cenocepacia*, these systems are involved in modulation of virulence-associated traits such as quorum sensing, biofilm formation, chemotaxis, and motility, as well as general physiological functions (Buroni et al., 2009; Bazzini et al., 2011a,b). A proteomic analysis of the effects of RND-4 gene deletion revealed about 70 differentially expressed proteins, most of which were associated with cellular functions other than drug resistance. This suggests that RND-4 plays a more general role in *B. cenocepacia*’s biology (Gamberi et al., 2013). Aside from the key role that eﬄux, especially RND-mediated eﬄux, plays in adaptation to antibiotic exposure (Bazzini et al., 2011b; Sass et al., 2011; Tseng et al., 2014), survival of *Burkholderia* species in various niche environments and accompanying conditions, e.g., the cystic fibrosis airways (Mira et al., 2011), marine habitats (Maravic et al., 2012), oxygen levels (Hemsley et al., 2014), exposure to noxious chemicals (Rushton et al., 2013), and other ecological niches (Liu et al., 2015), involves to various degrees changes in eﬄux pump expression.

## Burkholderia pseudomallei

### Eﬄux Pumps and Drug Resistance

**FIGURE 1:**
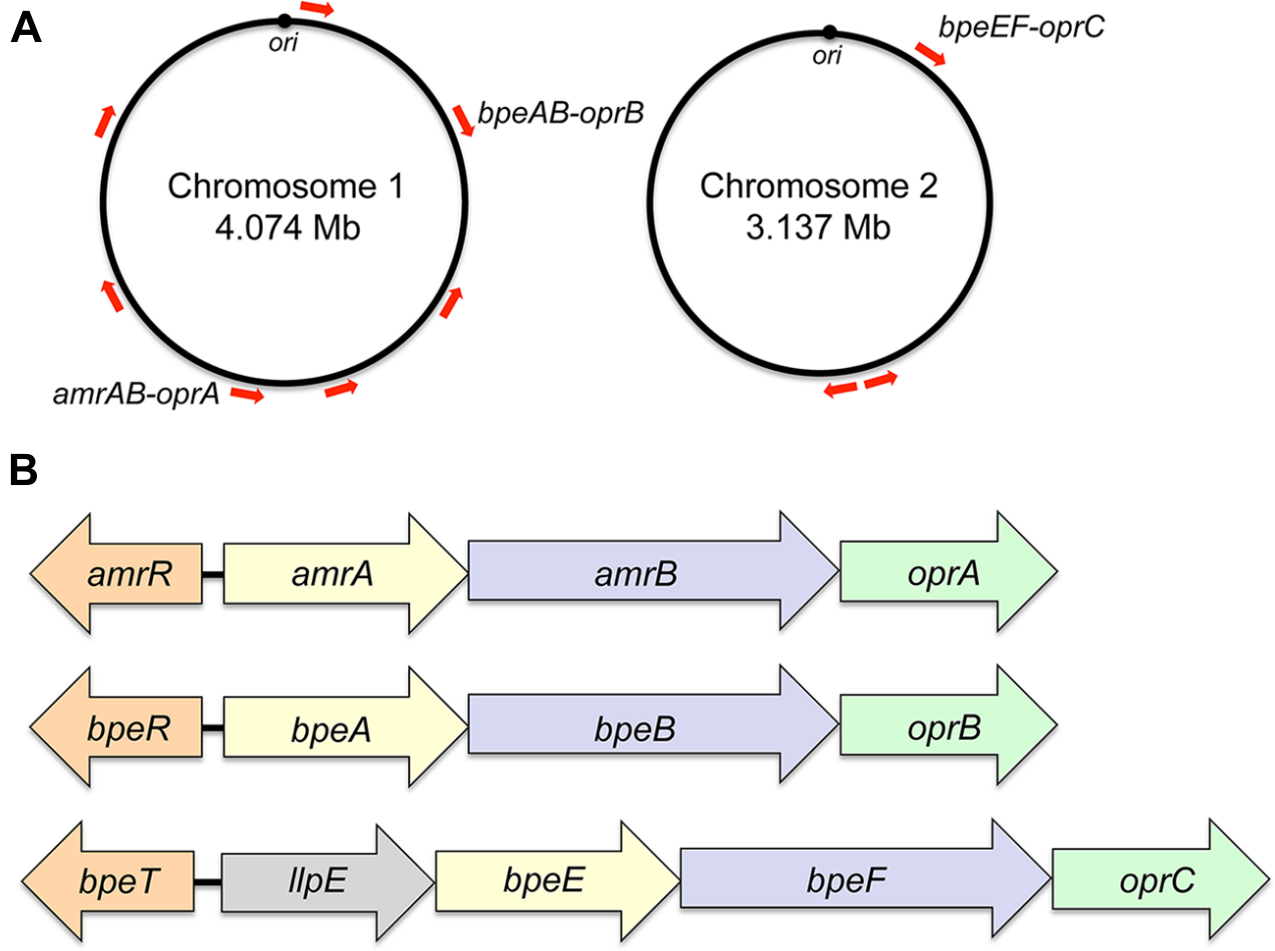
**Genomic location and operon organization of *Burkholderia pseudomallei* resistance nodulation cell division (RND) eﬄux pumps**. **(A)** Chromosomal locations of RND eﬄux operons in strain K96243. Red arrows indicate the approximate locations and transcriptional orientations of RND operons on chromosomes 1 and 2. Operons encoding characterized eﬄux pumps are labeled. Although RND operon distribution is conserved amongst diverse *B. pseudomallei* strains, locations on the respective chromosomes may vary because of chromosome rearrangements. The origins of replication (*ori*) in the two strain K96243 chromosomes are marked. Their respective locations on the chromosomes of other strains may vary because of chromosome rearrangements. **(B)** Transcriptional organization of characterized RND operons. The genes encoding the membrane fusion proteins (*amrA, bpeA, bpeE*), RND transporter (*amrB, bpeB, bpeF*) and outer membrane protein channel (*oprA*, *oprB*, *oprC*) are color-coded. The *bpeE–bpeF–oprC* genes are in the same operon as *llpE*, which is annotated as a putative lipase/carboxyl esterase. LlpE is not required for BpeEF-OprC eﬄux pump function. Orthologs of LlpE are present in all annotated operons that encode *bpeE–bpeF–oprC* orthologs. The *amrR* and *bpeR* genes encode repressors of *amrAB-oprA* and *bpeAB-oprB*, respectively. The *bpeT* gene encodes a LysR-type transcriptional regulator of *llpE*-*bpeE-bpeF-oprC*.

Initially, the presence of genomic DNA sequences in *B. pseudomallei* that hybridize with the multidrug resistance eﬄux gene *oprM* of *P. aeruginosa* was interpreted as evidence that eﬄux-mediated multidrug eﬄux systems may also be present in *B. pseudomallei* (Bianco et al., 1997). A recent survey of documented *B. pseudomallei* antibiotic resistance mechanisms indeed showed that eﬄux is the dominant mechanism affecting most classes of antibiotics (Schweizer, 2012b). Sequenced *B. pseudomallei* genomes encode numerous eﬄux systems, but as with other non-enteric bacteria only RND pumps have to date been shown to confer resistance to clinically significantly antibiotics. The K96243 and other *B. pseudomallei* genomes encode at least 10 RND systems, seven of which are encoded by chromosome 1 and three by chromosome 2 (Holden et al., 2004; **Figure 1A**). Although RND operon distribution is conserved amongst diverse *B. pseudomallei* strains, locations on the respective chromosomes may vary because of chromosome rearrangements. Bioinformatic analyses indicate that not all of the RND operons encode drug eﬄux pumps. For instance, one system seems to encode components of a general secretion (Sec) system. Although the presence of many RND systems can be detected in clinical and environmental isolates at the transcriptional (Kumar et al., 2008) and protein level (Schell et al., 2011), this expression is not necessarily linked to increased drug resistance. Meaningful studies to address their function are complicated because isogenetic progenitor and/or comparator strains are generally not available. Further hindering eﬄux pump characterization are select agent guidelines, which restrict certain methods, such as selection of spontaneous drug resistant mutants that may display altered eﬄux expression profiles. These investigations are now facilitated by the availability of several *B. pseudomallei* strains, for instance Bp82 (Propst et al., 2010), which are excluded from select agent rulings. To date three RND drug eﬄux pumps – AmrAB-OprA, BpeAB-OprB, and BpeEF-OprC – have been characterized in some detail (**Figure 1B**). There is evidence that small molecule compounds such as MC-207,110, phenothiazine antipsychotics, and antihistaminic drugs like promazine can be used to potentiate antibiotic efficacy, primarily by inhibition of RND eﬄux pumps (Chan et al., 2007b).

#### AmrAB-OprA

The AmrAB-OprA eﬄux pump was the first eﬄux pump described in *B. pseudomallei* (Moore et al., 1999). It is responsible for this organism’s high-level intrinsic aminoglycoside and macrolide resistance (Moore et al., 1999; Viktorov et al., 2008). Rare (∼1 in 1,000) naturally occuring aminoglycoside susceptible *B. pseudomallei* isolates have previously been identified (Trunck et al., 2009; Podin et al., 2014). They do not express the AmrAB-OprA pump either due to regulatory mutations (Trunck et al., 2009), point mutations affecting the AmrB RND transporter amino acid sequence (Podin et al., 2014), or because the entire *amrAB-oprM* operon is missing due to a genomic deletion (Trunck et al., 2009). Although the AmrAB-OprA eﬄux pump is expressed in prototype strains, exposure to antimicrobials can select for unknown mutations that cause its over-expression resulting in increased resistance. For instance, prototype strain 1026b is moderately susceptible [minimal inhibitory concentration (MIC) 4–8 μg/mL] to the ketolide cethromycin and exposure to this compound readily selects for highly resistant (MIC > 128 μg/mL) derivatives due to AmrAB-OprA over-expression (Mima et al., 2011). To date, AmrAB-OprA expression is the sole reported aminoglycoside and macrolide resistance mechanism observed in *B. pseudomallei*.

AmrAB is closely related to *P. aeruginosa* MexXY, which is expressed in some aminoglycoside resistant mutants and together with OprM constitutes a functional effux pump (Mine et al., 1999; Sobel et al., 2003; Morita et al., 2012). MexXY associates with the *mexAB-oprM* encoded OprM outer channel protein because the *mexXY* operons of most *P. aeruginosa* strains do not encode a cognate outer mebrane channel protein. However, some strains, e.g., *P. aeruginosa* PA7, encode a *mexAB-oprA* operon akin and functionally equivalent to *B. pseudomallei amrAB-oprA* (Morita et al., 2012).

#### BpeAB-OprB

The BpeAB-OprB eﬄux pump was first described in strain KHW from Singapore (Chan et al., 2004) and subsequently characterized in Thai strain 1026b (Mima and Schweizer, 2010). It is not significantly expressed in wild-type strains. BpeAB-OprB expression is regulated by BpeR and *bpeR* mutants exhibit low-level chloramphenicol, fluoroquinolone, tetracycline, and macrolide resistance (Chan et al., 2004; Chan and Chua, 2005; Mima and Schweizer, 2010). Although the original studies with strain KHW indicated a role of BpeAB-OprB in aminoglycoside resistance (Chan et al., 2004), these results could not be confirmed with strain 1026b (Mima and Schweizer, 2010). At present, the observed differences in BpeAB-OprB substrate spectrum between strains KHW and 1026b are not understood. Because of the low levels of resistance bestowed by BpeAB-OprB and naturally occuring antibiotic resistant BpeAB-OprB over-expressing mutants have yet to be identified, the clinical significance of this pump remains unclear.

Although BpeAB-OprB is related to *P. aeruginosa* MexAB-OprM (Poole et al., 1993; Li et al., 1995; Mima and Schweizer, 2010), the respective features are quite divergent. While *P. aeruginosa* MexAB-OprM is widely expressed and responsible for this bacterium’s intrinsic resistance to numerous antibacterial compounds (Poole et al., 1993; Li et al., 1995; Poole, 2001), *B. pseudomallei* BpeAB-OprB is not widely expressed and does seem to play only a minor role in this bacterium’s resistance to antimicrobials.

#### BpeEF-OprC

**FIGURE 2:**
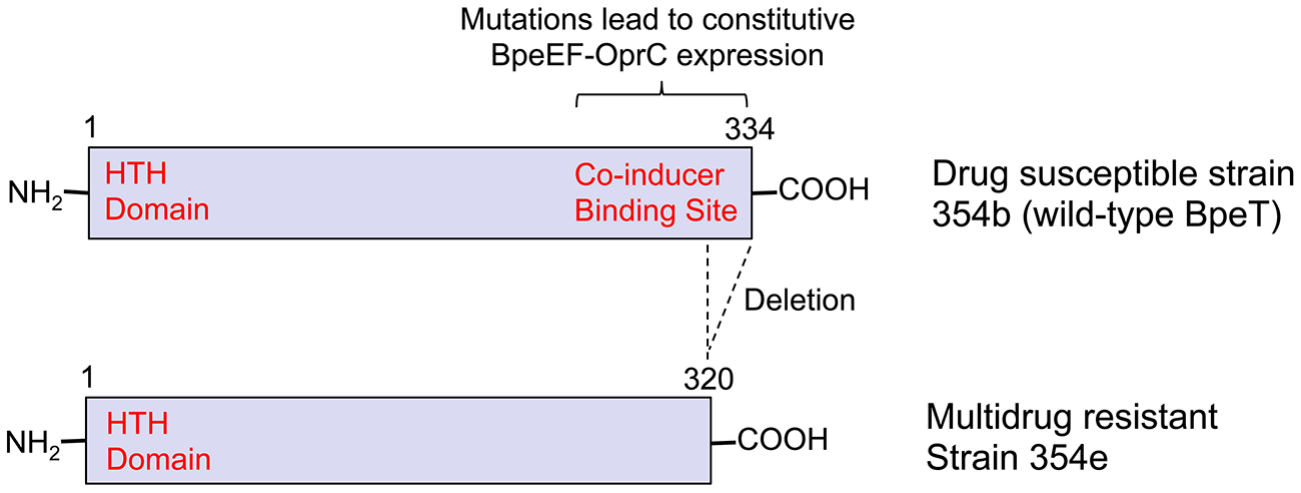
**The transcriptional regulator BpeT and locations of mutations causing multidrug resistance due to BpeEF-OprC expression**. The figure illustrates the cause of BpeEF-OprC expression leading to a multidrug resistance phenotype due to *bpeT* mutations by comparing a prototype strain, in this instance a primary melioidosis isolate (strain 354b; **Top**), and a relapse isolate (strain 354e; **Bottom**). Strain 354b **(Top)** expresses wild-type BpeT that exhibits the typical LysR-type regular organization with an amino-terminal helix-turn-helix (HTH) DNA-binding domain and a co-inducer binding site located within the carboxy-terminal half. Point mutations located within the latter domain result in constitutive BpeEF-OprC eﬄux pump expression. Strain 354e **(Bottom)** expresses BpeEF-OprC constitutively due to deletion of the last 14 native *bpeT* codons as a consequence of a 800 kb inversion affecting chromosome 2 (Hayden et al., 2012).

BpeEF-OprC was first identified as a chloramphenicol and trimethoprim eﬄux pump by expression in an eﬄux-compromised *P. aeruginosa* strain (Kumar et al., 2006). This pump is not expressed in *B. pseudomallei* wild-type strains, but only regulatory mutants. For instance, it is constitutively expressed in naturally occuring *bpeT* mutants (Hayden et al., 2012; **Figure 2**). When expressed, BpeEF-OprC confers high-level resistance to chloramphenicol, fluoroquinolones, tetracyclines, and trimethoprim. It is responsible for widespread trimethoprim resistance in clinical and environmental *B. pseudomallei* isolates (Podnecky et al., 2013). Pump expression is inducible by some pump substrates, which when present at sub MIC levels may lead to cross-resistance with other pump substrates (Schweizer, 2012a).

BpeEF-OprC is related to *P. aeruginosa* MexEF-OprN (Koehler et al., 1997), which shares properties such as substrate profiles and selection of pump-expressing regulatory mutants by chloramphenicol as previously demonstrated with both *P. aeruginosa* MexEF-OprN (Koehler et al., 1997) and *B. thailandensis* BpeEF-OprC (Biot et al., 2011).

### Other Functions of *B. pseudomallei* Eﬄux Pumps

Quorum sensing is an important determinant of virulence factor regulation in bacteria. Numerous studies with *P. aeruginosa* indicate the involvement of several RND pumps in quorum sensing and thus several virulence traits by being involved in transport of cell-to-cell signaling molecules and their inhibitors (Evans et al., 1998; Pearson et al., 1999; Koehler et al., 2001; Aendekerk et al., 2005; Hirakata et al., 2009; Tian et al., 2009). At least one eﬄux pump regulator, MexT, modulates expression of virulence factors, albeit independent of the function of the MexEF-OprN eﬄux pump whose expression it regulates (Tian et al., 2009). A *P. aeruginosa* MexAB-OprM deletion strain is also compromised in its ability to invade Madin–Darby canine kidney (MDCK) cells (Hirakata et al., 2009). Based on these findings with *P. aeruginosa*, several studies with *B. pseudomallei* explored the effects of eﬄux on quorum sensing and virulence. Studies with strain KHW showed that the BpeAB-OprB eﬄux pump was required: (1) for the secretion of the acyl homoserine lactones produced by this strains quorum-sensing systems (Chan et al., 2007a); and (2) secretion of several virulence-associated determinants, including siderophore and biofilm formation (Chan and Chua, 2005), but these observations could not be confirmed with strain 1026b (Mima and Schweizer, 2010). Cell invasion of and cytotoxicity toward human A549 lung epithelial and THP-1 macrophage cell were signifianctly reduced in a KHW BpeAB-deficient strain (Chan and Chua, 2005). Adherence to A549 cells and virulence in the BALB/c mouse intranasal infection model were not affected in the AmrAB-OprA deficient Bp340 mutant, a derivative of strain 1026b (Campos et al., 2013). BALB/c mouse intranasal infection studies also showed that in addition to AmrAB-OprA, BpeAB-OprB, and BpeEF-OprC were not required for virulence (Propst, 2011; Schweizer, 2012a). The AmrAB-OprA eﬄux pump is also not required for efficient killing of *Caenorhabditis elegans* by *B. pseudomallei* (O’Quinn et al., 2001). The BpeAB-OprB pump has been implicated in being involved in spermidine homeostasis in strain KHW with exogenous spermidine and *N*-acetylspermidine activating *bpeA* transcription (Chan and Chua, 2010).

## Eﬄux Pumps in other *Burkholderia* Species

### Burkholderia mallei

In part due to ongoing in host evolution of this obligate pathogen, *B. mallei* is generally more susceptible to antimicrobials than its progenitor *B. pseudomallei*. For instance, many *B. mallei* strains are susceptible to aminoglycosides. In the ATCC 23344 prototype strain this susceptibility results from a 50 kb chromosomal deletion encompassing the *amrAB-oprA* operon (Nierman et al., 2004). Strains NCTC10229 and NCTC10247 are likely aminoglycoside susceptible because only remnants of the *amrAB-oprA* operon are present (Winsor et al., 2008). Genes and operons encoding other eﬄux pumps, including BpeAB-OprB and BpeEF-OprC, are present but whether they encode functional eﬄux systems remains remains to be established.

### Burkholderia thailandensis

One study indicated the presence of an MFS eﬄux pump, with an associated regulatory protein of the multiple antibiotic resistance regulator (MarR) family (Grove, 2010). However, expression of the eﬄux pump was only responsive to urate, xanthine, and hypoxanthine and thus the significance of this transporter in *B. thailandensis*’ antibiotic resistance, if any, is unclear.

In contrast, the contributions of RND pumps to this bacterium’s antibiotic resistance have been established. It was shown that chloramphenicol exposure selects for expression of an RND eﬄux pump, BpeEF-OprC, that also extrudes fluoroquinolones, tetracycline, and trimethoprim (Biot et al., 2011). Doxycycline selection resulted in mutants that either over-expressed AmrAB-OprA or BpeEF-OprC, and exhibited the multidrug resistance profiles associated with expression of these eﬄux pumps (Biot et al., 2013). Mutational analysis of these mutants suggested that BpeAB-OprB could at least partially substitute for absence of either AmrAB-OprA or BpeEF-OprC. Unlike other Gram-negative bacteria, cell envelope properties, eﬄux pump repertoire, and resulting drug resistance profile make *B. thailandensis* suitable as a *B. pseudomallei* BSL-2 surrogate for drug efficacy studies (Schweizer, 2012c).

### Burkholderia vietnamiensis

Transposon mutagenesis studies aimed at identification of polymyxin B susceptible mutants identified a gene encoding a NorM multidrug eﬄux protein (Fehlner-Gardiner and Valvano, 2002). While *norM* expression in an *E. coli acrAB* deletion mutant complemented its norfloxacin susceptibility, its inactivation in *B. vietnamiensis* only affected susceptibility to polymyxin but not other antibiotics.

Unlike other *Burkholderia* species, including most BCC members, the majority of environmental and clinical *B. vietnamiensis* isolates are aminoglycoside susceptible (Jassem et al., 2011). Aminoglycoside resistance as a result of chronic infection or *in vitro* exposure to aminoglycosides is the result of the AmrAB-OprM eﬄux pump, which is most likely a homolog of *B. pseudomallei* and *B. thailandensis* AmrAB-OprA (Jassem et al., 2011, 2014). Of note is the observation that eﬄux pump expression in mutants that acquired resistance during infection was due to missense mutations in the *amrAB-oprM* regulator *amrR*, but not those mutants derived from antibiotic pressure *in vivo* (Jassem et al., 2014).

## Burkholderia Eﬄux Pump Mutants as Experimental Tools

The high-level intrinsic antibiotic resistance of many *Burkholderia* species complicates their genetic manipulation, use in studies of intracellular bacteria with the aminoglycoside protection assay, and drug efficacy studies. It has been shown that eﬄux-compromised mutants of *B. cenocepacia* and *B. pseudomallei* greatly facilitate genetic manipulation of these species, as well as cell invasion studies using the aminoglycoside protection assay (Dubarry et al., 2010; Hamad et al., 2010; Campos et al., 2013). Eﬄux-compromised strains of *B. thailandensis* and *B. pseudomallei* strains have also proved useful for study of the eﬄux propensity of novel antimicrobial compounds (Liu et al., 2011; Mima et al., 2011; Teng et al., 2013; Cummings et al., 2014).

## Conclusion

*Burkholderia* species are well adapted to life in diverse, often adversarial, environments including those containing antimicrobials. This adaptation is facilitated by large genomes that bestow on the bacteria the ability to either degrade or expel noxious chemicals. As a result, opportunistic infections by pathogenic members of this species are difficult to treat because of intrinsic antibiotic resistance and persistence in the presence of antimicrobials. Resistance is in large part attributable to eﬄux pump expression, mostly members of the RND family. While the last decade has seen significant progress in study of drug eﬄux in *Burkholderia* species, progress still lags significantly behind other opportunistic pathogens, e.g., *P. aeruginosa* and *A. baumannii*, where eﬄux pumps also play significant roles in intrinsic and acquired drug resistance.

There are some unique aspects of eﬄux systems in *Burkholderia* species that are without parallel and studies of these may shed light on unique physiological functions of eﬄux pumps in these organisms. For instance, the first gene in the *bpeEF-oprC* operon, *llpE*, is co-transcribed with the genes encoding the BpeEF-OprC eﬄux pump components (Nair et al., 2004, 2005). Its deletion neither affects eﬄux pump function nor specificity for known antibiotic substrates. It is highly conserved throughout the *Burkholderia* genus and found in all sequenced genomes (Winsor et al., 2008). Based on homology, LlpE probably is an enzyme of the alpha/beta hydrolase family, recently annotated as a putative lipase/carboxyl esterase, and its conservation throughout the genus suggests an adaptation or survival benefit in a niche environment. The unique association of this enzyme with BpeEF-OprC and its role in *Burkholderia* biology warrant further studies of this enzyme.

When reviewing the *B. cenocepacia* eﬄux pump literature it becomes evident that eﬄux pump nomenclature in this species, especially that of the RND family is non-uniform and confusing (for instance CeoAB-OpcM, RND-1 to RND-16, BCA gene names, Mex1, orf, etc.), which makes comparisons with other Gram-negative bacteria unnecessarily cumbersome. As with other Gram-negative bacteria, the nomenclature initiated in *B. cenocepacia* by Dr. Jane Burns’ laboratory in the early 2000s, i.e., CeoAB-OpcM (Nair et al., 2004), would make the most sense and it is a pity that it was not followed in subsequent studies.

## Author Contributions

NP, KR, and HS contributed to all aspects of the work, including, but not limited to, conception and design, acquisition and analysis of data, writing the manuscript, and final approval of the version to be published.
